# An Emotion Assessment of Stroke Patients by Using Bispectrum Features of EEG Signals

**DOI:** 10.3390/brainsci10100672

**Published:** 2020-09-25

**Authors:** Choong Wen Yean, Wan Khairunizam Wan Ahmad, Wan Azani Mustafa, Murugappan Murugappan, Yuvaraj Rajamanickam, Abdul Hamid Adom, Mohammad Iqbal Omar, Bong Siao Zheng, Ahmad Kadri Junoh, Zuradzman Mohamad Razlan, Shahriman Abu Bakar

**Affiliations:** 1 Faculty of Electronic Engineering Technology, Universiti Malaysia Perlis (UniMAP), Arau 02600, Perlis, Malaysia; wenyean0412@gmail.com (C.W.Y.); iqbalomar@unimap.edu.my (M.I.O.); wendy880806@gmail.com (B.S.Z.); 2 Faculty of Electrical Engineering Technology, Universiti Malaysia Perlis (UniMAP), Arau 02600, Perlis, Malaysia; wanazani@unimap.edu.my (W.A.M.); abdhamid@unimap.edu.my (A.H.A.); 3 Department of Electronics and Communication Engineering, Kuwait College of Science and Technology, Doha Area, 7th Ring Road, Kuwait City 13133, Kuwait; m.murugappan@gmail.com; 4 School of Electrical and Electronic Engineering, Nanyang Technological University (NTU), 50 Nanyang Avenue, Singapore 639798, Singapore; yuva2257@gmail.com; 5 Institute of Engineering Mathematics, Universiti Malaysia Perlis, Arau 02600, Perlis, Malaysia; kadri@unimap.edu.my; 6 Faculty of Mechanical Engineering Technology, Universiti Malaysia Perlis (UniMAP), Arau 02600, Perlis, Malaysia; zuradzman@unimap.edu.my (Z.M.R.); shahriman@unimap.edu.my (S.A.B.)

**Keywords:** emotion, stroke, electroencephalogram (EEG), bispectrum

## Abstract

Emotion assessment in stroke patients gives meaningful information to physiotherapists to identify the appropriate method for treatment. This study was aimed to classify the emotions of stroke patients by applying bispectrum features in electroencephalogram (EEG) signals. EEG signals from three groups of subjects, namely stroke patients with left brain damage (LBD), right brain damage (RBD), and normal control (NC), were analyzed for six different emotional states. The estimated bispectrum mapped in the contour plots show the different appearance of nonlinearity in the EEG signals for different emotional states. Bispectrum features were extracted from the alpha (8–13) Hz, beta (13–30) Hz and gamma (30–49) Hz bands, respectively. The k-nearest neighbor (KNN) and probabilistic neural network (PNN) classifiers were used to classify the six emotions in LBD, RBD and NC. The bispectrum features showed statistical significance for all three groups. The beta frequency band was the best performing EEG frequency-sub band for emotion classification. The combination of alpha to gamma bands provides the highest classification accuracy in both KNN and PNN classifiers. Sadness emotion records the highest classification, which was 65.37% in LBD, 71.48% in RBD and 75.56% in NC groups.

## 1. Introduction

Stroke is one of the highest causes of death in Malaysia, with more than 40,000 survivors are managing their health today [1]. Globally, there were 6.2 million deaths caused by stroke in 2017, where the highest rates of stroke mortality countries were reported in Eastern Europe, Africa, and Central Asia [2]. Stroke is caused by the insufficient supply of oxygen to the brain, damaging damage brain cells. This is turn will definitely affect some brain functions which results in stroke survivors to have difficulties in daily living, such as mobility, communication and expressing their thoughts. Also, stroke patients often suffer from emotional and behavioral changes due to their dissatisfaction with the current conditions.

Past studies have been carried out to investigate emotional changes in stroke patients as the influence of their physiological phenomenon [3,4,5]. These studies revealed that emotions and thoughts are seen as interactive reactions and are intimately related to the health and physiological problems. This leads to the increase risk of a second or recurrent stroke with persistent depression. Therefore, emotion recognition of stroke patients is very helpful in the diagnosis of their psychological and physiological conditions.

The assessment of the emotional conditions and mood of stroke patients is required during rehabilitation to identify the presence of mental health problems such as persistent depression and mood disorders. This also assists in identifying the severity of associated functional impairment of the patients.

Conventionally, emotion assessment can be done through interviews with patients [3,6], an observation on patients’ behaviors [6] as well as using standardized measures such as the Hospital Depression and Anxiety Scale (HADS) [6] and Beck Depression Inventory (BDI) [7]. These standardized measures determine the emotional states of the patient by scoring. However, the conventional approaches could be cheated by patients and information that is acquired then will be not accurate. Consequently, researchers tried other approaches to understand the emotional states of patients. Recent studies have stated that emotion assessment can be performed using physiological signals [8], such as skin conductance (SC) [9], respiration signal [10], electrocardiogram (ECG) [11], and electroencephalogram (EEG) [12].

This paper is organized as follows: Section 1, the problem and the context of the study in emotion assessment in stroke patients is discussed. Then, Section 2 reviews the literature on EEG analysis and non-linear features. This section also discusses the use of bispectrum features in EEG analysis. Section 3 focuses on the materials and methods used in this study, including the description of the EEG data, preprocessing, feature extraction, statistical analysis and classification methods. The next section presents the results and their discussions. Lastly, the summary of this paper is briefly discussed.

## 2. Related Works

EEG is the brain signal that can be measured from placing electrode sensors along the scalp to record the electrical activity of the brain that happens near the surface of the scalp [13]. EEG signal can be used for diagnostic purposes and any abnormalities detected from it connotes that there is brain disorder in that person.

From previous research, the brain has been reported as having higher responsibility and involvement in emotional activities [14]. The brain as the center of emotions is responsible to give responses when it perceives a stimulus. Hence, brain signals are able to provide emotional information of a stroke patient. With this concept, most recent studies of emotion assessment for stroke patients have utilized brain signals. Adamaszek et al. studied the emotional impairment using the event-related potentials (ERP) of a stroke patient [15], Doruk et al. studied the emotional impairment in stroke patients by comparing the emotional score in the Stroke Impact Scale (SIS) with the EEG features, the EEG power asymmetry and coherence [16]. Bong et al. assessed the emotions of stroke patients by using EEG signals in the time-frequency domain [12]. In this study, the electroencephalogram (EEG)-based emotion recognition algorithm is proposed to study the emotional states of stroke patients.

According to previous studies, stroke patients suffer from emotional impairment and consequently, hence their emotional experiences are less significant compared to normal people [15,17,18,19]. In another related study by Bong et al. [12], left brain damage (LBD) stroke patients were most dominant in perceiving sadness emotion and RBD stroke patients were most dominant in anger emotion. In addition, the authors’ dominant frequency band was the beta band by using wavelet packet transform (WPT) with Hurst exponent feature. The highest accuracy obtained was 76.04% in the happiness emotion for the normal control (NC) group using the features from beta to gamma band.

Previous emotional classification studies by Yuvaraj et al. [20] have also shown that the accuracy of the feature extracted from the single frequency band was higher in the beta and gamma bands. Also, the highest accuracy was obtained when all frequency bands from delta to gamma were combined. The author obtained an average accuracy of 66.80% in NC using the combinations of the five frequency bands. In addition, they obtained an average accuracy of 64.73% with the beta band and 65.80% with the gamma band of NC. These results were optimized with the application of the feature selection technique.

The brain is a chaotic dynamical system [21,22,23], where EEG signals are generated by nonlinear deterministic processes. This is also referred to as deterministic chaos theory with nonlinear coupling interactions between neuronal populations [24,25]. In contrast with linear analysis, nonlinear analysis methods will give more meaningful information about the emotional state changes of stroke patients. Over the last few years, a number of research works have been reported on analyzing EEG signals by using non-linear methods [25,26,27]. For example, a recurrence measure was applied to study the seizure EEG signals [25]. Zappasodi et al. used fractal dimension (FD) to study the neuronal impairment in stroke patients [26]. In addition, Acharya et al. studied sleep stage detection in EEG signals by using different nonlinear dynamic methods, higher order spectra (HOS) features and recurrence quantification analysis (RQA) features [28]. In their study, HOS was used to extract momentous information which helped with the diagnosis of neurological disorders.

HOS has been claimed as an effective method for analyzing EEG signals. HOS feature has been the most commonly used nonlinear feature. It is the frequency domain or spectral representation of higher order cumulants of a random process. HOS only includes cumulants with third order and above. HOS gains its advantage with the elimination of Gaussian noise and provides a high signal to noise ratio (SNR) [29,30]. HOS provides the ability to extract information deviation from Gaussian and preserves the phase information of signals. Thus, HOS is able to estimate the phase of the non-Gaussian parametric signals. In addition, HOS detects and characterizes nonlinearities in signals. In contrast, the second order measure is power spectrum, which can only reveal linear and Gaussian information of signals.

The third order HOS is bispectrum and is able to preserve phase information of EEG signals. Bispectrum is the easiest HOS to be worked out [31]. Bispectrum has been utilized in the emotional study in EEG signals. Yuvaraj et al. applied bispectrum to study the difference between Parkinson’s disease patients and normal people in six discrete emotions (happiness, sadness, fear, anger, surprise, and disgust) [27,32]. Hosseini applied bispectrum to classify the two emotional states (calm and negatives states) of normal subjects [33].

However, the emotional states of stroke patients are yet to be analyzed using the bispectrum features. Hence, in this work, the bispectrum feature is used to classify stroke patients’ EEG signals in different emotional states.

Bispectrum is proven in its ability to detect quadratic phase coupling (QPC), a phenomenon of nonlinearity interaction in EEG signals. QPC is the sum of phases at two frequency variables given by $f_{1}+f_{2}$ [34,35]. Bispectrum can be estimated through two approaches: direct and indirect methods. For a stationary, discrete time, random process x(k), the direct method is estimated by taking the 1D-Fourier transform of the discrete series given by:

(1) $$Bi\left(f_{1},f_{2}\right)=E\left[X\left(f_{1}\right)X\left(f_{2}\right)X^{*}\left(f_{1}+f_{2}\right)\right],$$

where *Bi* is the bispectrum magnitude, *E* [ ] denotes statistical expectation operation, X(f) is the Fourier transform (1-D FFT) of the time series, x(k) and * denote the complex conjugate.

For the indirect method, bispectrum is estimated by first estimating the third order cumulants of the random process x(k). Then the *n*^th^-order moment is equal to the expectation over the process multiplied by the (*n* − 1) lagged version of itself. Therefore, the third order moment, $m_{3x}$ is:

(2) $$m_{3x}\left(\tau_{1},\tau_{2}\right)=E\left[X\left(k\right)X\left(k+\tau_{1}\right)X\left(k+\tau_{2}\right)\right],$$

where *E* [ ] denotes statistical expectation operation, $\tau_{1}\;\mathrm{and}\;\tau_{2}$ are lags of the moment sequence.

The third order cumulant sequence, $C_{3x}\left(\tau_{1},\tau_{2}\right)$, is identical to its third order moment sequence. It can be calculated by taking an expectation over the process multiplied by 2 lagged versions given by:

(3) $$C_{3x}\left(\tau_{1},\tau_{2}\right)=E\left[X\left(k\right)X\left(k+\tau_{1}\right)X\left(k+\tau_{2}\right)\right].$$

The bispectrum, $B\left(f_{1},f_{2}\right),$ is the 2D-Fourier transform of the third order cumulant function is given by:

(4) $$B\left(f_{1},f_{2}\right)=\;\;\overset{\infty }{\underset{\tau_{1}=-\infty }{\sum }}\overset{\infty }{\underset{\tau_{2}=-\infty }{\sum }}C_{3x}\left(\tau_{1},\tau_{2}\right)\exp\left[-j\left(f_{1}\tau_{1}+f_{2}\tau_{2}\right)\right],\;\;\;$$

for $|f_{1}|\leq \pi$, $|f_{2}|\leq \pi$, and $|f_{1}+f_{2}|\leq \pi$.

**Figure 1:**
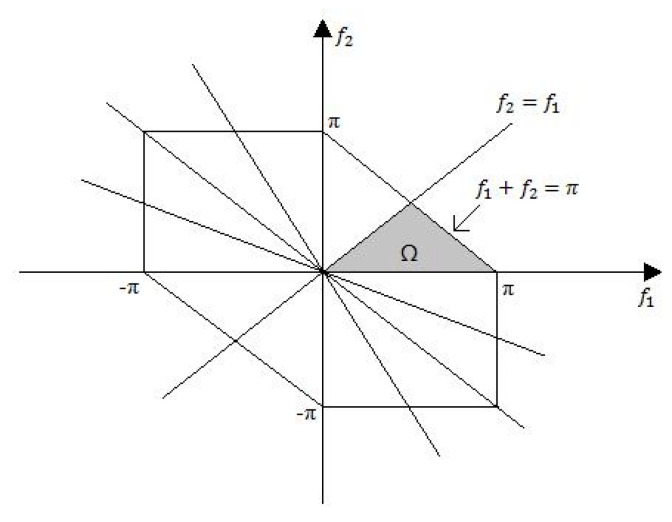
Symmetry regions and non-redundant region (Ω) of bispectrum.

Bispectrum is a symmetric function as shown in Figure 1. The shaded area is the non-redundant region of computation of the bispectrum, where $f_{2}\geq 0,\;f_{2}\geq f_{1},\;f_{1}+f_{2}\leq \pi$, which is sufficient to describe the whole bispectrum [36].

## 3. Materials and Methods

### 3.1. EEG Data

The EEG database used in this study was collected from stroke patients, with left brain damage (LBD), right brain damage (RBD) and normal control (NC) at the Hospital Canselor Tuanku Muhriz (HCTM), Kuala Lumpur. (formal approval obtained from UKM Medical Center and Ethics committee for human research, reference no.: UKM 1.5.3.5/244/FF-354-2012). The EEG raw signals of 15 subjects each from every group (LBD, RBD, and NC) were used for the analysis. The background and neurophysiological characteristics of the subjects in the three groups are described in Table 1. The subjects passed the Mini-Mental State Examination (MMSE) with scores of more than 24 over a total of 30 points which was conducted to exclude dementia. The subjects also passed the Beck Depression Inventory (BDI) with scores of less than 18 points, to exclude subjects with psychological problems. The Edinburg Handedness Inventory (EHI) was used to determine the handedness of the subjects, and measured in a scale from −1 to 1. The scales were interpreted as pure left hander for a score of −1, mixed left hander for a score of −0.5, neutral for a score of 0, mixed right hander for a score of 0.5 and pure right hander for a score of 1. From Table 1, the scores show that all subjects were right handers. All subjects were self-reported to have normal vision or corrected to normal vision (with spectacles or contact lenses) to ensure better effect of perceiving emotions from audio–visual stimuli.

**Figure 2:**
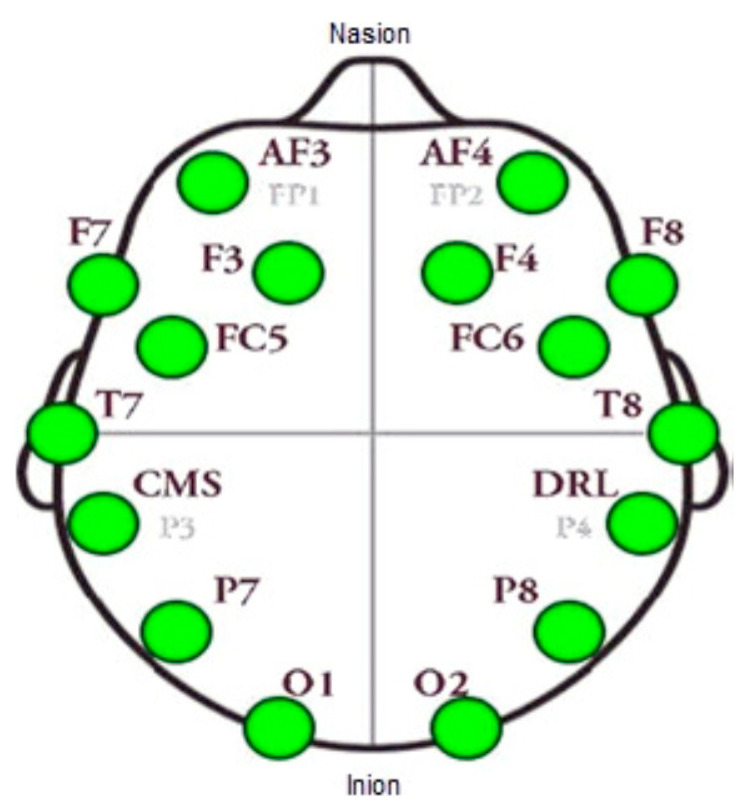
Electrodes placement of Emotiv EPOC according to 10–20 system.

The EEG data were collected using a 14-channel wireless EEG device, Emotiv EPOC headset, with built in digital 5th order Sinc filter. The electrode placement was based on the international standard 10–20 system as shown in Figure 2. The EEG data were collected at sampling frequency of 128 Hz. One of the limitations of the EEG is that it has poor spatial resolution as compared to high resolution brain imaging devices, such as functional magnetic resonance imaging (fMRI) and positron emission tomography (PET) scans [37]. However, the Emotiv EPOC device has 14 electrodes with 2 references providing appropriate spatial resolution as well as practical in terms of time and money for this study. Moreover, the EEG device provides high temporal resolution data that record the neural activity changes in milliseconds, which is impossible for fMRI and PET scans.

**Figure 3:**
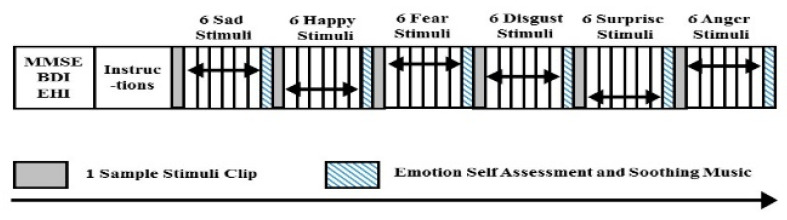
Data collection protocol.

To collect the emotional EEG data, an emotional elicitation protocol was designed to stimulate the emotional states of subjects. The data collection protocol is shown in Figure 3. The stimuli used to evoke the emotions in subjects were audio–visual in the form of video clips edited from International Affective Picture System (IAPS) and International Affective Digital Sound (IADS). Six emotional content video clips were presented to stimulate six discrete emotions, namely anger (A), disgust (D), fear (F), happiness (H), sadness (S) and surprise (SU) [12].

Prior to the experiment, the subjects were asked to complete the MMSE, BDI, and EHI tests and informed consent was given to them. Then, instruction about the experimental procedure was given to the subjects. The experiment started with a sample video clip, followed by six trials of video clips which were displayed continuously. Emotional EEG signals were recorded during the six trials video clips display. After that, the EEG recording was stopped for self-assessment, where the subjects were asked about the emotions they felt or perceived from the video clips. The self-assessment time was at least 1 min and it was subject-dependent. During this period, subjects were asked to relax and get ready for the next video. This was to avoid stimulus order effects. Then the experiment began with the sadness emotion. The same experimental procedures were repeated for happiness (H), fear (F), disgust (D), surprise (SU) and anger (A) emotions. There were a total of 42 video clips, including those of the sample video clips. The duration of each video clip was 46 s to 1 min, therefore, the total duration of the data collection was between 90 to 120 min.

### 3.2. Preprocessing

There were a total of 36 trials of EEG signals collected from each subject in all groups (LBD, RBD and NC). The collected EEG signals were preprocessed to remove the effects of noises and artifacts that caused interference to the raw signals. The preprocessing of the EEG signals was performed using MATLAB.

The artifacts due to eye blinks were filtered using thresholding method, where the potentials higher than 80 µV and lower than −80 µV were offset from each EEG raw signal [32]. A 6th order Butterworth bandpass filter was used to filter the EEG signals with cut-off frequencies from 0.5 to 49 Hz to extract the delta to gamma frequency bands [32].

### 3.3. Feature Extraction

The indirect method was used in this study to estimate bispectrum using the *bispeci* function in the MATLAB Higher Order Statistics Toolbox. The number of points used to form each fast Fourier transform (NFFT) was 1024. The bispectrum features were extracted from data by using 50% overlap with Hanning window. The preprocessed time domain EEG data were segmented into six seconds length for every channel. Each data segment is also known as an epoch, and contains 768 data. Three types of EEG frequency sub-bands were used for analysis, namely the alpha (8–13) Hz, beta (13–30) Hz and gamma (30–49) Hz bands.

Bispectrum features were computed from the non-redundant region (Ω) of bispectrum The features extracted from each epoch were variance (v), sum of logarithmic of bispectrum (H1), sum of logarithmic of diagonal elements in the bispectrum (H2), first moment of diagonal elements in the bispectrum (H3), second moment of diagonal elements in the bispectrum (H4) and moment of bispectrum (H5).

The variance, v of the bispectrum was computed as:

(5) $$v=\frac{1}{N-1}\overset{N}{\underset{i=1}{\sum }}{\left|B_{i}-\mu \right|}^{2},$$

where *N* is the total number of bispectrum in Ω, μ is the mean of bispectrum in Ω, $B_{i}$ is the bispectrum series for i = 1, 2, 3, …, *N*.

Sum of logarithmic amplitudes of bispectrum (*H*1):

(6) $$H1=\underset{\Omega }{\sum }\log\left(\left|B\left(f_{1},f_{2}\right)\right|\right),$$

where Ω is the non-redundant region of bispectrum, $f_{1}$ and $f_{2}$ are frequency variables of bispectrum and $B\left(f_{1},f_{2}\right)$ is the bispectrum feature of $f_{1}$ and $f_{2}$ in Ω.

The sum of logarithmic amplitudes of diagonal elements in the bispectrum (*H*2):

(7) $$H2=\underset{\Omega }{\sum }\log\left(\left|B\left(f_{m},f_{m}\right)\right|\right),$$

where Ω is the non-redundant region of bispectrum and $B\left(f_{m},f_{m}\right)$ is the diagonal element of bispectrum feature in Ω.

The first-order spectral moment of amplitudes of diagonal elements in the bispectrum (*H*3):

(8) $$H3=\overset{N}{\underset{m=1}{\sum }}m\cdot \log\left(\left|B\left(f_{m},f_{m}\right)\right|\right),$$

where $B\left(f_{m},f_{m}\right)$ is the diagonal element of bispectrum feature in Ω, *N* is the total number diagonal elements of bispectrum in Ω, and m = 1, 2, 3, …, *N*.

The second-order spectral moment of the amplitudes of diagonal elements in the bispectrum (*H*4):

(9) $$H4=\overset{N}{\underset{m=1}{\sum }}{\left(m-H3\right)}^{2}\cdot \log\left(\left|B\left(f_{m},f_{m}\right)\right|\right),$$

where $B\left(f_{m},f_{m}\right)$ is the diagonal element of bispectrum feature in Ω, *N* is the total number diagonal elements of bispectrum in Ω, and m = 1, 2, 3, …, *N*.

The moment of bispectrum (*H*5):

(10) $$H5=\left(\sqrt{f_{1}^{2}+f_{2}^{2}}\right)\cdot \left|B\left(f_{1},f_{2}\right)\right|,$$

where $f_{1}$ and $f_{2}$ are frequency variables of bispectrum and $B\left(f_{1},f_{2}\right)$ is the bispectrum feature of $f_{1}$ and $f_{2}$ in Ω.

### 3.4. Statistical Analysis

One-way analysis of variance (ANOVA) was used to test the significant difference of the bispectrum features among the six emotions classes for LBD, RBD and NC respectively. The use of ANOVA was to statistically analyze the bispectrum features for whether there were differences among the class means of the six emotions. The use of ANOVA requires the assumption that the observations from the feature were approximately normally distributed, the observations were independent and the variances of the classes were equal. The null hypothesis was: “All the emotions of the extracted feature have equal mean”. The null hypothesis was rejected and the bispectrum features were validated as statistically significant among the six emotional states if the *p*-value is less than or equal to 0.05. When the null hypothesis was not rejected, it implies that all the emotions of the extracted feature have equal mean, thus the feature that failed to reject the null hypothesis was not suitable to be used for emotion classification.

### 3.5. Classification

Each feature used for classification has a total of 90 trials (6 trials × 15 subjects) with 84 feature vectors (14 channels × 6 windows) for each emotion. The *k*-nearest neighbor (KNN) and probabilistic neural network (PNN) classifiers were used to classify the six emotions in the three groups (LBD, RBD and NC). The KNN is one of the most widely applied classifier due to it lower complexity and fast decision making. The KNN searches for the nearest distance or to examine for the most likeliness between the unknown sample and the training dataset. The distance of the unknown sample and the training dataset is determined by the distance metric. In this study, the Cityblock distance metric was implemented in the KNN classification [38].

The PNN uses the Parzen window for nonparametric approximation of the probability distribution function (PDF) of each class and applies Bayes’ rule to allocate the new input data to the class with the highest probability by using the PDF of each class [39]. The classifier parameter is the spread value and is proportional to the standard deviation of the Parzen window in PNN. A small spread value gives narrow PDF, whereas a large spread value gives wide PDF and the classifier becomes less selective [40,41].

In this work, the k values of 1 to 15 were tested in KNN and the spread values of 0.1 to 1.5 with an increment of 0.1 were used in PNN to classify the features. The performance of the classifiers was validated through 10-fold cross validation, where 90% of the data were used for training and 10% of the data were used for testing.

## 4. Results and Discussions

**Figure 4:**
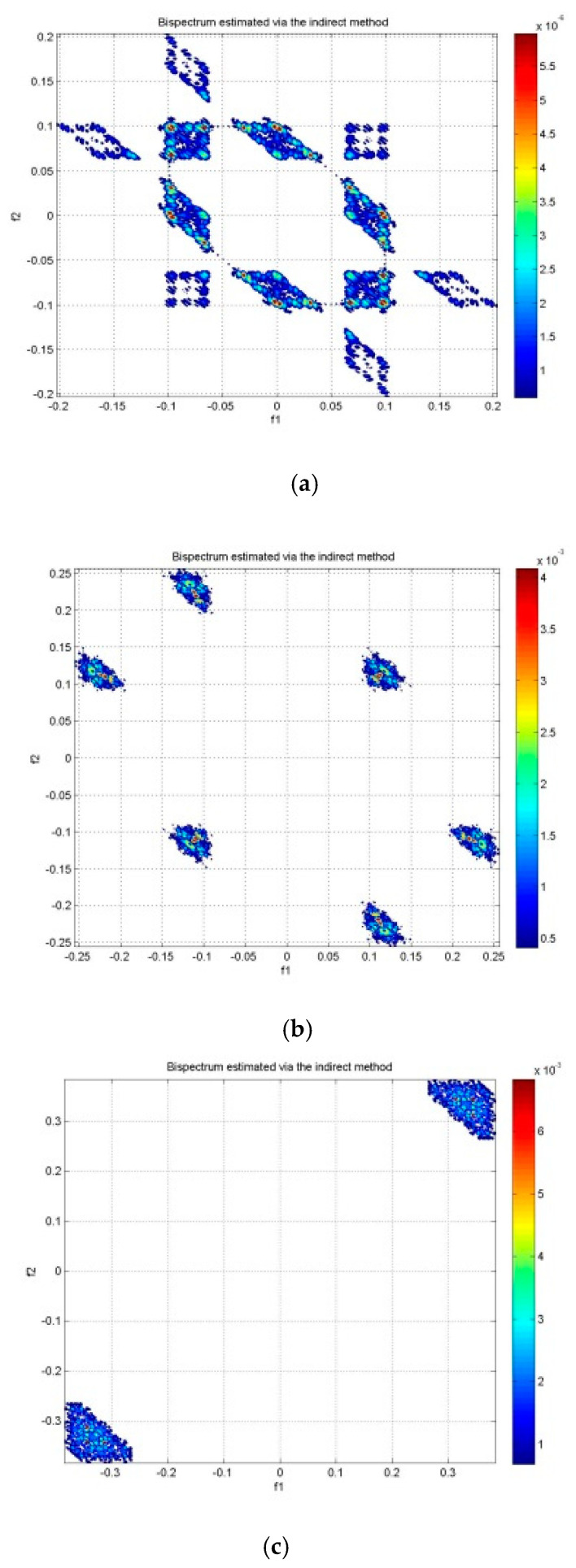
Bispectrum contour plot of LBD anger emotion in (**a**) alpha band, (**b**) beta band, and (**c**) gamma band.

Bispectrum features were extracted from the EEG signals in three groups of subjects (LBD, RBD, and NC) for the analysis of six emotions, namely anger (A), disgust (D), fear (F), happiness (H), sadness (S), and surprise (SU). The contour plots of the estimated bispectrum using the anger emotion of one subject from the LBD group was plotted for the alpha, beta and gamma bands as shown in Figure 4. The plots of the bispectrum magnitude show the relationship between the two bispectrum frequency variables, $f_{1}$ and $f_{2}$, of the anger emotion. In Figure 4, the $f_{1}$ (x-axis) and $f_{2}$ (y-axis) are phased coupled. Frequency variables that are phase coupled indicate the presence of quadratic phase coupling (QPC) [31], where the QPC represents the underlying neuronal interaction of the emotional state at the frequencies ($f_{1}$ and $f_{2}$). The higher magnitude indicates stronger QPC between the frequencies. The red color represents the greatest increase in the magnitude of bispectrum, while the blue color represents the greatest decrease in the magnitude of bispectrum.

The distribution of the bispectrum over the ($f_{1}$, $f_{2}$) plane differs in each frequency band. The alpha band in Figure 4a shows more bispectrum distribution at lower phase coupled frequencies, which is between (0.04, 0.04) Hz and (0.1, 0.1) Hz in the non-redundant region and other symmetry regions. Whereas the beta band in Figure 4b and gamma band in Figure 4c show the bispectrum distribution at higher phase coupled frequencies. These are between (0.1, 0.1) Hz and (0.2, 0.2) Hz in the beta band and between (0.3, 0.3) Hz and (0.4, 0.4) Hz in the gamma band. Moreover, the maximum magnitude of the bispectrum in the alpha band is the lowest among the three frequency bands. The beta band has larger maximum bispectrum magnitude than the alpha band, while the gamma band has the largest maximum bispectrum magnitude among the frequency bands.

**Figure 5:**
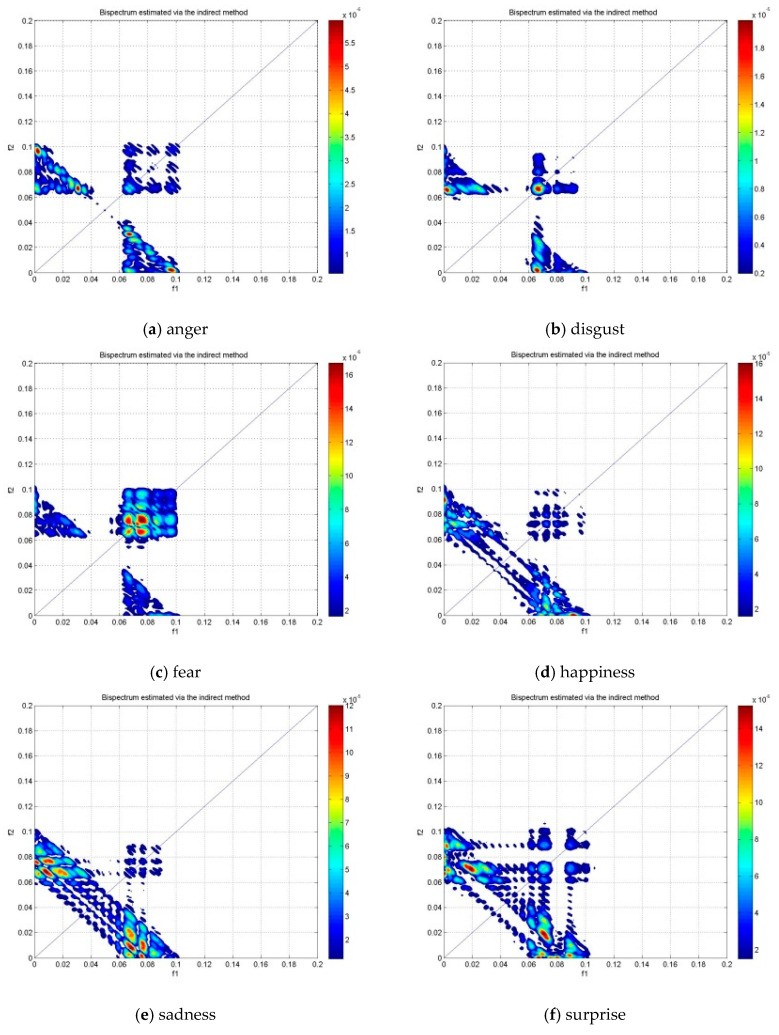
The bispectrum contour plot of the non-redundant region and one symmetry region in the alpha band of subject #1 NC group.

**Figure 6:**
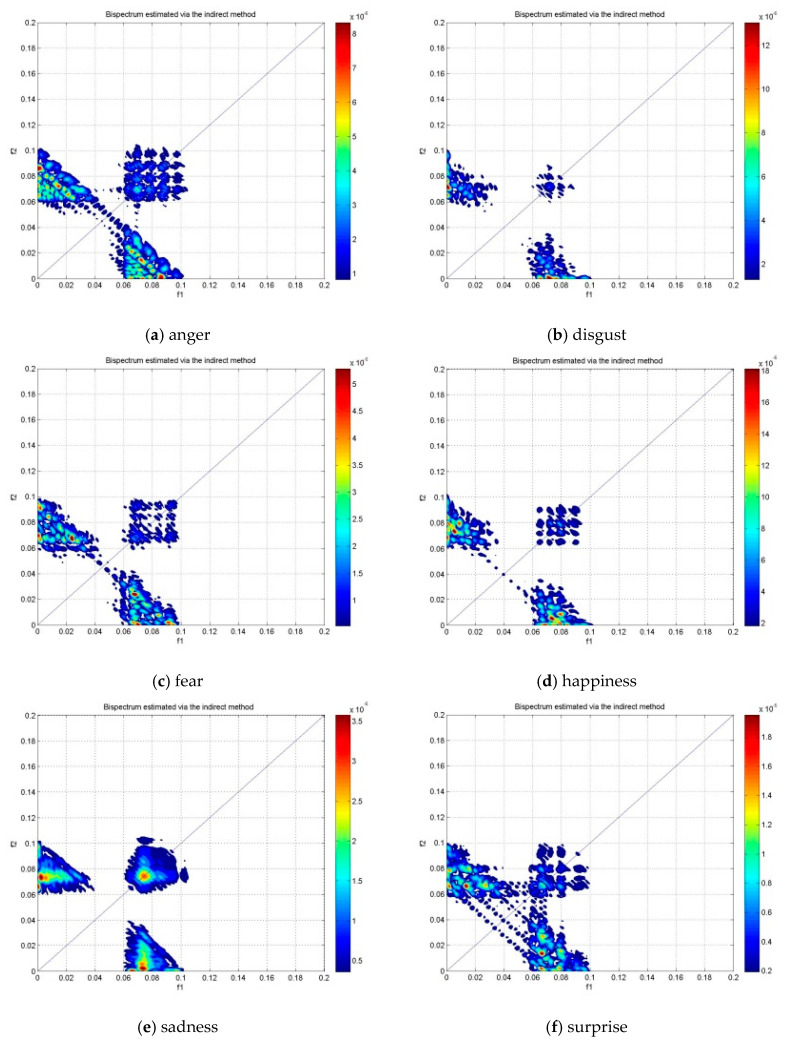
The bispectrum contour plot of the non-redundant region and one symmetry region in the alpha band of subject #1 LBD group.

**Figure 7:**
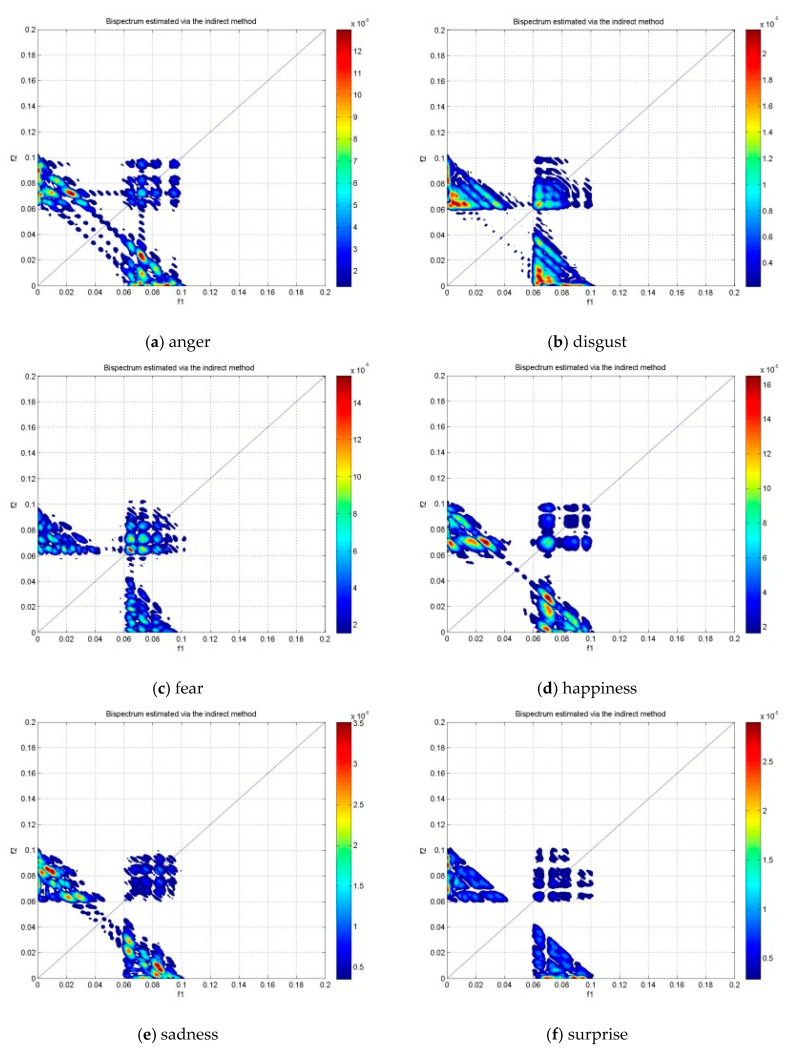
The bispectrum contour plot of the non-redundant region and one symmetry region in the alpha band of subject #1 RBD group.

Figure 5, Figure 6 and Figure 7 show the bispectrum plots in the non-redundant region and one symmetry region of the six emotions of Subjects #1 from NC, LBD and RBD groups, respectively. From these figures, the different emotional states have different bispectrum distribution over the plane with different phased coupled peaks and maximum magnitude for each. In the past studies, bispectrum has been claimed as a useful signal classification method as it is able to show distinctive distribution in different conditions, such as the left-hand motor imagery and the right-hand motor imagery [42]. The bispectrum provides an EEG feature that is able to recognize these two conditions. Another study has shown that the bispectrum feature is different before meditation and during meditation [43]. This study revealed that the bispectrum exhibit more phase-coupled distribution during meditation than the state before meditation. In addition, the maximum bispectrum magnitude increased during meditation. For the non-human experiment, the “induced’’ ischemic stroke in rat showed the difference bispectrum distribution in different states of ischemia [44]. In this study, the bispectrum distribution decreased as the rat turns from normal state to ischemic state. Consequently, the distinctive bispectrum pattern of the six emotional states presented in this study implies that the emotional states of each group were distinguishable by applying bispectrum analysis. The significant difference of the emotional states using the bispectrum feature is further validated by the statistical analysis using ANOVA as shown in Table 2.

From the experiment, six types of bispectrum features were extracted from preprocessed EEG data of LBD, RBD and NC. The statistical test using ANOVA was performed on the extracted features and the degrees of freedom were 45,354. The results are shown in Table 2 in three different frequency bands for LBD, RBD and NC respectively. For a *p*-value less than or equal to 0.05, this indicates that the differences between some of the means of the emotional states are statistically significant. The significant bispectrum features imply that there is an interaction of neuronal subcomponents at different frequencies in different emotional states. The shaded *p*-values show the feature which are statistically not significant between the means of emotion classes as they are larger than 0.05. All the bispectrum features were statistically significant in LBD, RBD and NC except the second moment of the diagonal elements in bispectrum (*H*4), thus, *H*4 was discarded in classification. Moreover, from Table 2, the F values are higher in *H*1 and *H*3 for LBD, *H*2 and *H*5 in RBD and *v*, *H*5, and *H*2 in NC. The highest overall F values are in the LBD group, while NC has comparably smaller values compared to both LBD and RBD groups.

**Figure 8:**
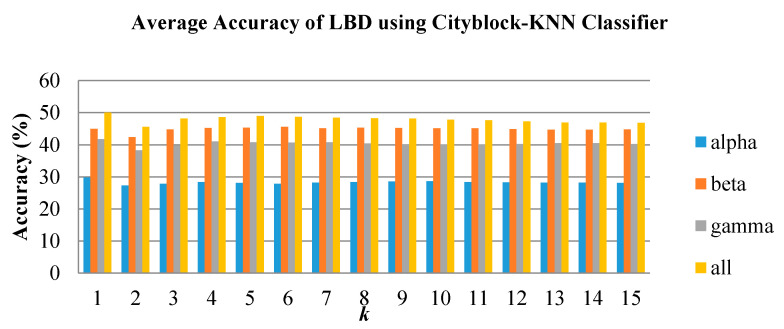
Average classification performance of bispectrum features by varying *k* values for the LBD group using *k*-nearest neighbor (KNN).

**Figure 9:**
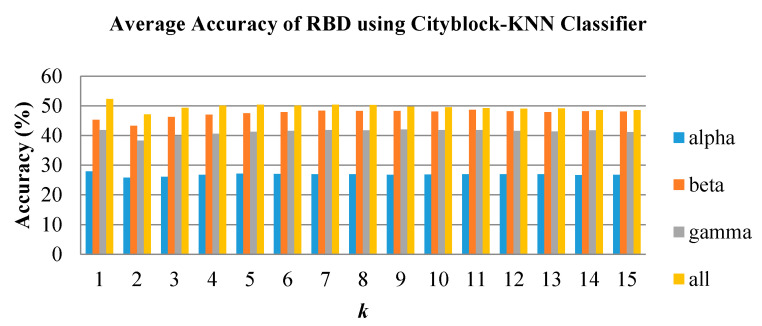
Average classification performance of bispectrum features by varying *k* values for the RBD group using KNN.

**Figure 10:**
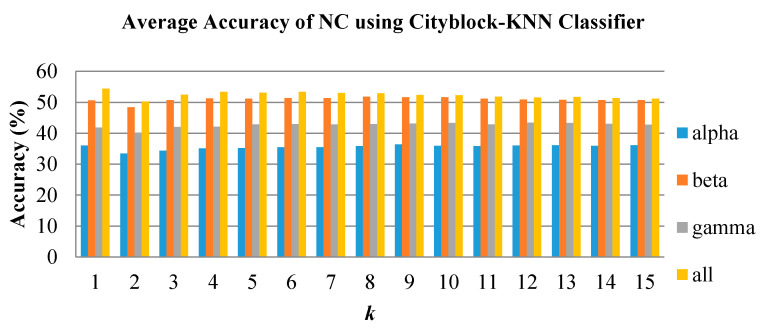
Average classification performance of bispectrum features by varying *k* values for the NC group using KNN.

In emotion classification, the features were trained with varying k values for KNN and spread values for PNN. The classifiers were tested for all groups and frequency bands. Figure 8, Figure 9 and Figure 10 show the classification performance of varying *k* values in three individual EEG frequency sub-bands (alpha, beta, and gamma) and the combination of the three bands using Cityblock KNN classifier. From the figures, the average accuracy of the bispectrum features was similar across all *k* values tested for alpha, beta, and gamma bands. However, the *k* value of 1 achieved the highest average accuracy when using the features from the combination of the alpha to gamma band. Moreover, the combination of the alpha to gamma band significantly performs better than other frequency bands for all *k* values as shown in Figure 8, Figure 9 and Figure 10. The beta band, on the other hand, is the single band that performs best among the three EEG sub-bands.

**Figure 11:**
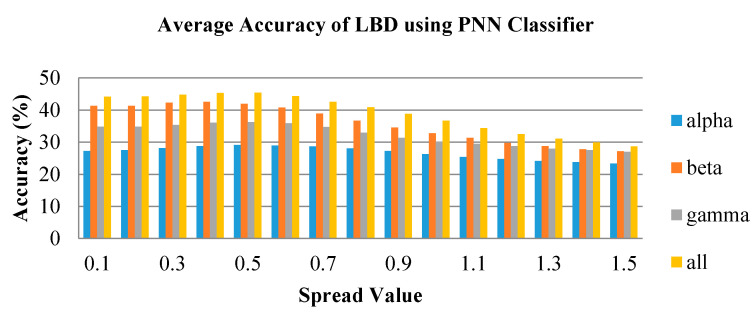
Average classification performance of bispectrum features by varying spread values for the LBD group using probabilistic neural network (PNN).

**Figure 12:**
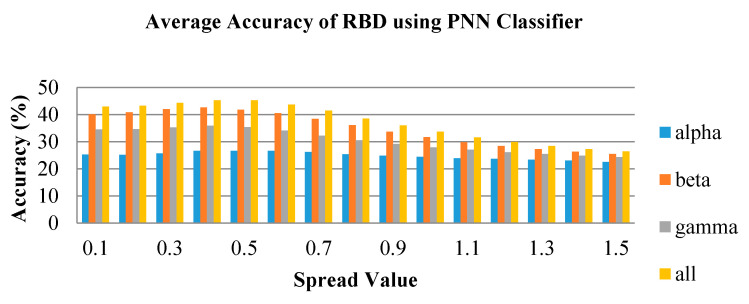
Average classification performance of bispectrum features by varying spread values for the RBD group using PNN.

**Figure 13:**
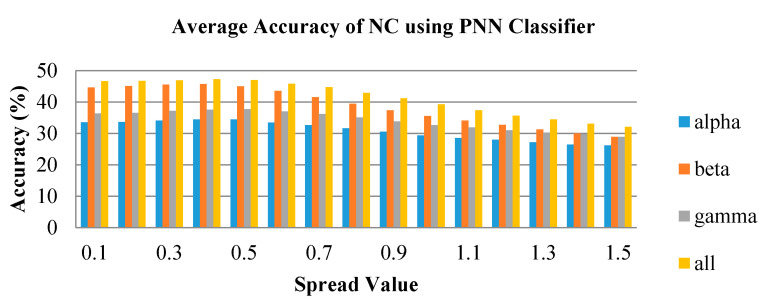
Average classification performance of bispectrum features by varying spread values for the NC group using PNN.

In Figure 11, Figure 12 and Figure 13, the average accuracy of the emotion classification of varying spread values using PNN classifier are plotted for LBD, RBD and NC, respectively. For most of the features, the accuracies are consistent at the spread value between 0.1 and 0.6. Then the accuracy is observed to gradually drop when spread value is larger than 0.6 and further declines when spread value increases. The spread value of 0.4 was chosen to classify the six emotional states as it has achieved the optimum accuracy for most of the features. Similarly, the combination of frequency bands has the highest average accuracy for all spread values. The beta frequency band is the best performance individual frequency band among the three frequency sub-bands in all groups.

Table 3 shows the average accuracy of all emotional states of the bispectrum features from the combination of all bands from alpha to gamma using KNN and PNN classifiers. For both classifiers, the optimum parameters for LBD, RBD and NC are found to be the same. The optimum *k* value in KNN classification for all three groups is 1, and the optimum spread values in PNN classification is 0.4. In Table 3, the KNN is observed to have higher average accuracy than the PNN classifier for all three groups. Notably, the *H*3 feature is seen to have the highest average accuracy feature in all three groups using KNN, whereas the *H*3 feature achieves the highest accuracy in the LBD group using PNN. Meanwhile, the *H*1 feature obtains highest average accuracy in RBD and NC using PNN. According to the results obtained, the top three features are H3, *H*1, and *H*2 for all the groups, whereas the worst performing bispectrum feature is the variance. The highest average classification accuracy is 65.40% in the NC group using KNN. Hence the *H*3 feature is considered the most effective bispectrum feature in this study.

The confusion matrix of the *H*3 feature in KNN emotion classification is presented in Table 4, Table 5 and Table 6 for LBD, RBD and NC, respectively. From Table 4, the highest predicted class is happiness in LBD. In Table 5, the RBD group has the highest predicted value in sadness and surprise emotions, whereas the NC group has the highest predicted value in sadness emotion in Table 6. For PNN classification, the confusion matrix is presented in Table 7, Table 8 and Table 9 for each subject group. Likewise, the PNN classification predicted the happiness emotion correctly in the LBD group, as shown in Table 7. In addition, the sadness emotion has the highest classification accuracy in RBD and NC groups as shown in Table 8 and Table 9.

**Figure 14:**
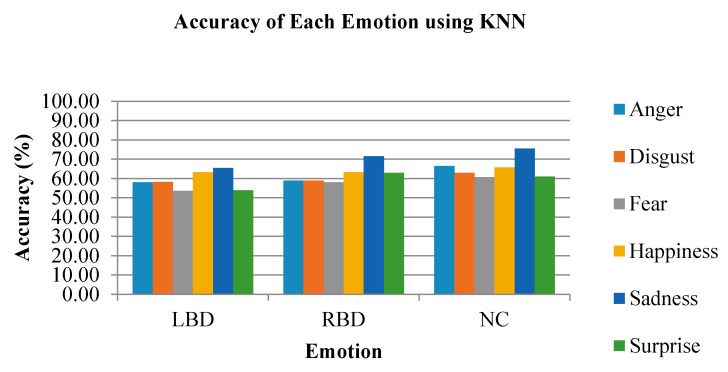
The accuracy of each emotional state using the KNN classifier.

The classification rates using the KNN classifier for individual emotions are show in Figure 14. From the figure, the emotion with highest accuracy in all groups was sadness, where the LBD group achieved 65.37%, RBD group achieved 71.48% and NC group achieved 75.56%. Meanwhile, the fear emotion recorded the lowest accuracy in all the three groups, which was 53.52%, 57.96% and 60.74% for LBD, RBD and NC respectively.

**Figure 15:**
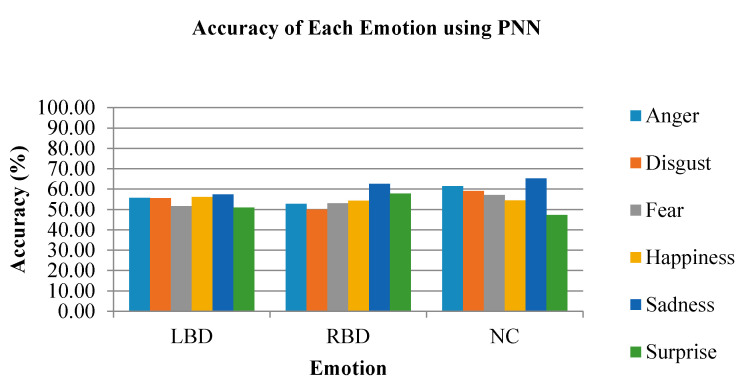
The accuracy of each emotional state using the PNN classifier.

The accuracy of the individual emotional states classified using the PNN classifier is shown in Figure 15. The PNN classifier has the same highest accuracy emotion with KNN, which was the sadness emotion. The LBD group has 57.41%, RBD has 62.59% and NC has 65.19% classification accuracy for sadness emotion using PNN. In Figure 15, the lowest classification accuracy for LBD and NC groups is the surprise emotion, which is 50.93% and 47.22%, respectively. In the RBD group, on the other hand, disgust emotion recorded the lowest classification accuracy of only 50.00%.

In this work, the surprise and fear achieved lower recognition rates compared to other emotions. The happiness was the most accurately recognized emotion, as well as the facial expressions for anger, sadness and disgust [45,46]. According to past studies, there is no convincing evidence for the surprise and fear emotions to be accurately recognized [47,48,49].

The emotional state that shows highest classification accuracy in each group (LBD, RBD and NC) indicates that the emotion is more significant compared to other emotional states in the respective groups. From this current result, all of the LBD, RBD and NC groups show highest classification accuracy for sadness. Meanwhile, NC group exhibits the highest average accuracy for both classifiers, followed by RBD with LBD trailing behind.

As a result, in this study, the LBD and RBD stroke patients have recorded a lower classification accuracy compared to the NC. This suggests that the emotional states of NC are more significant than the stroke patients. In order to validate the significant differences among the three groups, ANOVA was used to test the statistical difference among the average accuracy obtained from the KNN classifier and the resultant *p*-value was less than 0.05. Hence, the emotion classification accuracy for LBD, RBD and NC were statistically significant. This signifies the significant difference among the three groups, which implies that there are differences in the emotional experiences between LBD, RBD and NC groups. From this work, the NC group was observed to have the highest emotion classification accuracy, followed by the RBD group and the LBD group performed worst. Therefore, the NC group has the highest efficiency in EEG emotional classification with the use of machine learning, while the LBD group the lowest.

This work is significant to those past studies in which only second order measures of statistics, such as the power spectrum [50,51], which is a linear feature, was used. The power spectrum can only reveal the amplitude information about the EEG signals, the phase information, such as the phase coupling in the signal, cannot be observed by applying the power spectrum. Furthermore, the use of linear approaches has ignored the nonlinear characteristics of the EEG signals, thus, bispectrum was implemented in this study to detect and characterize the nonlinearities of EEG signals. Also, this current study using the bispectrum was able to provide distinctive information for different emotional states which was useful for emotion classification by achieving the highest accuracy of 75.56% using the H3. bispectrum feature.

## 5. Conclusions

The importance of emotion assessment of stroke patients stems from the need to seek information on the severity of emotional impairment symptoms. Therefore, an accurate emotion assessment approach is required to identify the symptoms of mood disorders in stroke patients. This work proposed the use of the bispectrum feature to classify the discrete emotions (anger, disgust, fear, happiness, sadness and surprise) of stroke patients and normal people. This study aims to develop an accurate emotion identification method, which can be used to recognize the current emotional state of strokes patient during diagnosis.

In this work, the bispectrum reveals the presence of QPC in the EEG signals and exhibits different QPC relations in each emotional state. This difference in the harmonic components and peaks were shown in the bispectrum contour plots arising from the nonlinear interactions between neuronal populations in each emotional state. In this study, the proposed method of emotion classification by using the bispectrum feature and KNN classifier has shown its effectiveness in the combination of alpha to gamma frequency bands. In addition, the bispectrum feature, H3, was able to provide an accuracy of 75.56% in the NC group. Moreover, the proposed method gave a comparable result with some current studies in emotion classification, However, there were only six types of bispectrum features implemented in this study and there are more to be explored. Also, future works could also focus on the optimization of the classification accuracy.

To conclude, bispectrum-based features are effective to analyze the nonlinearity EEG signals, and therefore is a useful feature for emotion assessment. Bispectrum feature was able to provide the emotional information of stroke patients and hence can be used as the substitute for conventional observation-based or scoring methods.

## Figures and Tables

**Table 1:** Background and neurophysiological characteristics (mean ± std) of left brain damage (LBD), right brain damage (RBD), and normal control (NC) subjects.

Variables	LBD	RBD	NC	*p*-Value
Sample size, *N*	15	15	15	NA
Age (year)	56.73 ± 6.15	55.87 ± 6.21	51.87 ± 4.19	0.05979
Female/male	5/10	5/10	12/3	NA
MMSE score (range: 0–30)	26.93 ± 1.77	27.2 ± 1.87	28.87 ± 0.81	0.00392
BDI scale (range: 0–21)	7.07 ± 2.91	6.20 ± 1.90	5.47 ± 1.20	0.14936
EHI (range: −1 to 1)	0.85 ± 0.50	0.85 ± 0.47	0.89 ± 0.40	0.96493
Duration of disease (year)	1.74 ± 1.47	2.24 ± 1.97	NA	NA

**Table 2:** Statistical validation results of LBD, RBD, and NC by using ANOVA (statistically significant at *p* ≤ 0.05).

Group	Features	Alpha		Beta		Gamma	
		F Value	*p*-Value	F Value	*p*-Value	F Value	*p*-Value
LBD	*v*	11.403	<0.001	8.081	<0.001	28.332	<0.001
	*H*1	49.542	<0.001	37.080	<0.001	36.839	<0.001
	*H*2	35.788	<0.001	48.353	<0.001	78.123	<0.001
	*H*3	35.993	<0.001	50.731	<0.001	78.527	<0.001
	*H*4	1.716	0.127	1.347	0.241	1.595	0.158
	*H*5	27.171	<0.001	31.501	<0.001	41.921	<0.001
RBD	*v*	3.091	0.009	18.464	<0.001	9.743	<0.001
	*H*1	11.070	<0.001	4.255	0.001	10.508	<0.001
	*H*2	18.778	<0.001	14.958	<0.001	28.436	<0.001
	*H*3	18.529	<0.001	15.113	<0.001	26.585	<0.001
	*H*4	1.785	0.112	8.005	<0.001	2.676	0.020
	*H*5	7.429	<0.001	27.299	<0.001	25.434	<0.001
NC	*v*	6.256	<0.001	14.068	<0.001	8.138	<0.001
	*H*1	2.993	0.011	6.004	<0.001	10.874	<0.001
	*H*2	4.887	<0.001	20.415	<0.001	38.428	<0.001
	*H*3	4.903	<0.001	20.139	<0.001	36.136	<0.001
	*H*4	2.335	0.040	0.442	0.820	1.550	0.171
	*H*5	6.704	<0.001	11.123	<0.001	13.184	<0.001

**Table 3:** Summary of average accuracy of all emotions of different bispectrum features using the combination of alpha to gamma bands.

Classifier	KNN			PNN		
Group	LBD	RBD	NC	LBD	RBD	NC
*k*/Spread	1	1	1	0.4	0.4	0.4
*v*	31.02	30.28	32.72	28.43	28.30	28.70
*H*1	58.27	61.79	63.67	53.24	**55.06**	**57.41**
*H*2	55.59	60.06	62.90	49.88	49.72	55.25
*H*3	**58.67**	**62.22**	**65.40**	**54.57**	52.84	56.11
*H*5	46.54	47.38	47.10	40.46	40.31	38.83

**Table 4:** Confusion matrix of the LBD group using the *H*3 feature in KNN classification.

		Actual					
		A	D	F	H	S	SU
Predicted	A	35	8	4	3	2	8
	D	4	31	6	3	5	5
	F	5	5	31	3	2	2
	H	3	2	7	41	7	3
	S	3	3	5	4	35	5
	SU	4	5	1	0	3	31

**Table 5:** Confusion matrix of the RBD group using the *H*3 feature in KNN classification.

		Actual					
		A	D	F	H	S	SU
Predicted	A	36	3	5	3	0	3
	D	2	35	4	3	2	4
	F	4	10	32	3	6	3
	H	3	4	9	36	5	4
	S	1	1	2	6	38	2
	SU	8	1	2	3	3	38

**Table 6:** Confusion matrix of the NC group using the *H*3 feature in KNN classification.

		**Actual**					
		**A**	**D**	**F**	**H**	**S**	**SU**
Predicted	A	39	4	1	4	1	6
	D	2	40	3	0	3	9
	F	1	4	34	4	3	4
	H	2	3	6	38	5	0
	S	2	1	6	6	41	1
	SU	8	2	4	2	1	34

**Table 7:** Confusion matrix of the LBD group using the *H*3 feature in PNN classification.

		Actual					
		A	D	F	H	S	SU
Predicted	A	32	6	3	4	1	7
	D	8	32	7	3	4	1
	F	5	5	30	3	4	2
	H	5	4	5	37	7	7
	S	3	2	6	5	35	3
	SU	1	5	3	2	3	34

**Table 8:** Confusion matrix of the RBD group using *H*1 feature in PNN classification.

		Actual					
		A	D	F	H	S	SU
Predicted	A	27	4	0	1	1	6
	D	5	35	5	2	4	5
	F	7	3	32	8	4	1
	H	4	2	6	34	2	5
	S	1	2	6	6	41	2
	SU	10	8	5	3	2	35

**Table 9:** Confusion matrix of the NC group using the *H*1 feature in PNN classification.

		Actual					
		A	D	F	H	S	SU
Predicted	A	32	3	2	2	1	8
	D	5	35	10	4	6	8
	F	5	3	33	2	4	3
	H	4	5	5	35	3	2
	S	3	2	3	9	40	3
	SU	5	6	1	2	0	30
